# Improved Machine Learning Predictions of EC50s Using Uncertainty Estimation from Dose–Response Data

**DOI:** 10.1021/acs.jcim.5c00249

**Published:** 2025-05-19

**Authors:** Hugo Bellamy, Joachim Dickhaut, Ross D. King

**Affiliations:** † Department of Chemical engineering and biotechnology, 2152 University of Cambridge, Cambridge CB2 1TN, United Kingdom of Great Britain and Northern Ireland; ‡ 5184 BASF, Ludwigshafen 67056, Germany

## Abstract

In early-stage drug design, machine learning models often rely on compressed representations of data, where raw experimental results are distilled into a single metric per molecule through curve fitting. This process discards valuable information about the quality of the curve fit. In this study, we incorporated a fit-quality metric into machine learning models to capture the reliability of metrics for individual molecules. Using 40 data sets from PubChem (public) and BASF (private), we demonstrated that including this quality metric can significantly improve predictive performance without additional experiments. Four methods were tested: random forests with parametric bootstrap, weighted random forests, variable output smearing random forests, and weighted support vector regression. When using fit-quality metrics, at least one of these methods led to a statistically significant improvement on 31 of the 40 data sets. In the best case, these methods led to a 22% reduction in the root-mean-squared error of the models. Overall, our results demonstrate that by adapting data processing to account for curve fit quality, we can improve predictive performance across a range of different data sets.

## Graphical abstract

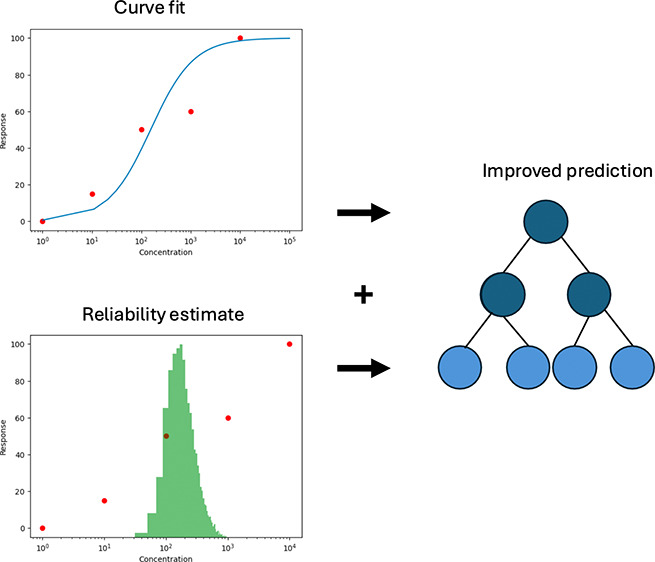

## Introduction

Quantitative structure activity relationship (QSAR) models are used to identify high-activity molecules for drug design and other bioactivity problems.^1,2^ Based on previously collected experimental data, models are constructed to predict the activity of untested molecules. While these models are often a useful tool in drug design processes,^3–5^ they are restricted by the quality of the available data.^6,7^ In this work, we focus on QSAR data sets where the relative quality of different data points varies significantly, and demonstrate that incorporating information about data point quality into the modeling process leads to improved predictions.

Our experiments used data sets containing dose–response experiments for different molecules. These experimental results are converted to EC50 values via a curve fitting procedure. An EC50 is the concentration at which a molecule induces a response of 50% of the maximum effect and is a commonly used metric in small molecule drug design.^8^ While EC50s are useful metrics, calculating reliable values from experimental data can be challenging.^9^ Figure 1 illustrates this challenge, showing examples of both a reliable (a) and unreliable (b) fit of EC50 curves.

**Figure 1:**
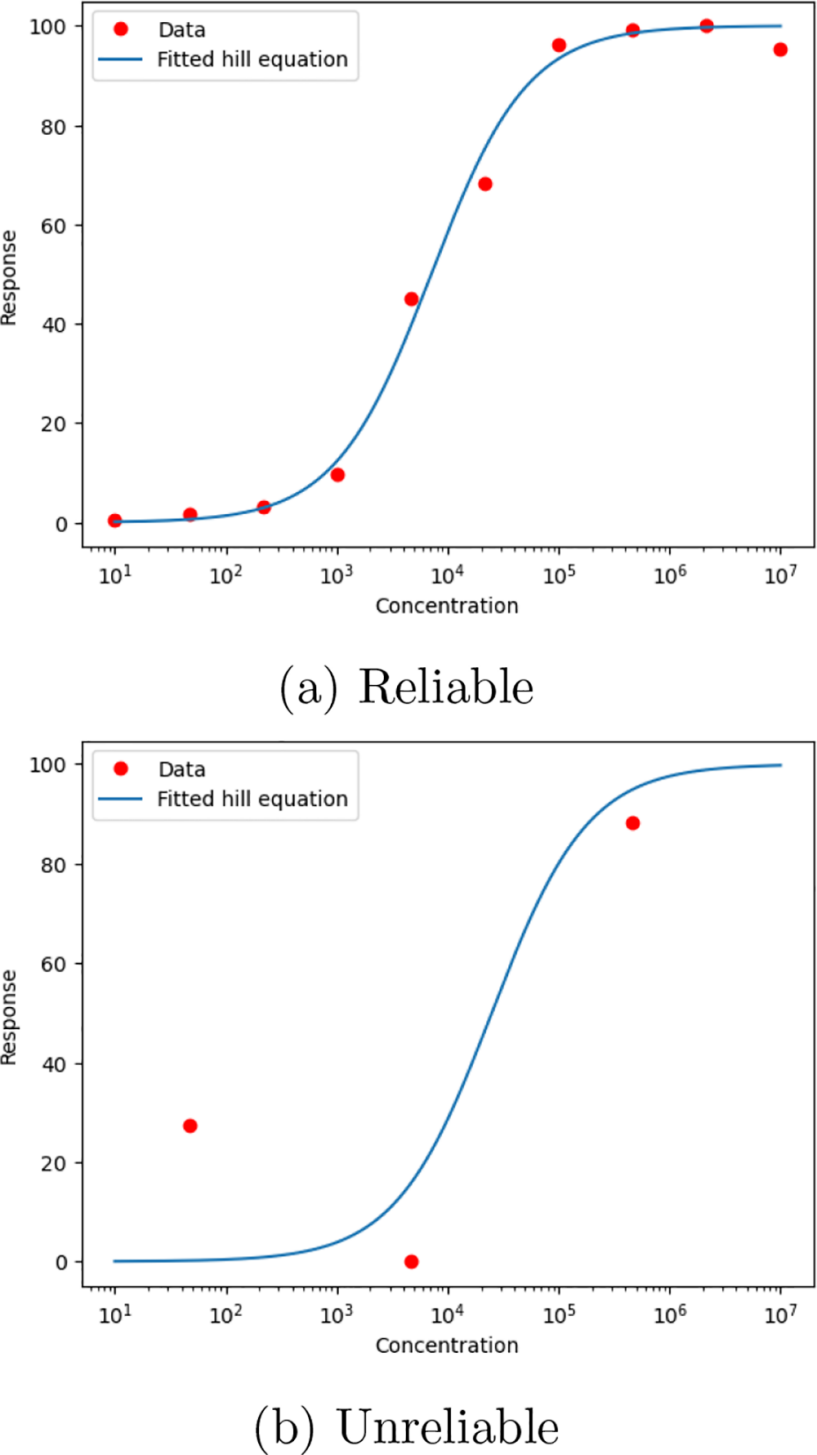
(a) Reliable vs (b) unreliable fit of the Hill equation to experimental data points.

The standard recommendations are to ignore data points for which a reliable EC50 cannot be established. However, not only can this approach introduce bias, but it results in experimental data being ignored by the model. Systematic bias can occur because molecules with very low or very high EC50s can often end up with unreliable fits. For example, a common practice is to test all molecules at the same set of concentrations (as is the case in all of the PubChem data sets used in our study) and to discard calculated EC50 values that do not have two measurements above and below the EC50.^10^ This would result in excluding molecules with either very high or very low activity.

Rather than discarding data with uncertain fits, we propose incorporating information about fit quality directly into the QSAR modeling process. During curve fitting, alongside the EC50, we calculate a “fit-quality metric”, a numerical score reflecting how closely the mathematical curve matches the experimental data points. Lower scores indicate a better fit and thus a more reliable EC50. This fit-quality metric helps us to estimate the uncertainty in each EC50 value, quantifying how confident we are in the calculated EC50. A poor fit (high fit-quality score) suggests high uncertainty – meaning the experimental data does not strongly pinpoint a single EC50 value. Conversely, a good fit (low fit-quality score) suggests low uncertainty, increasing our confidence that the calculated EC50 is close to the true biological value.

We used this fit quality metric while training modified machine learning methods. Compared to standard approaches, which ignore fit quality - using only the EC50s during model fitting, these methods showed significant performance improvements on most of the data sets tested. This approach was particularly motivated by BASF data sets in which EC50 quality varies substantially between molecules tested against the same target, due to measurement noise and varying numbers of experiments per molecule (as illustrated in Figure 1).

Our experiments use random forests and support vector machines for modeling, both popular methods that have been applied across different domains. For example, drug discovery,^11–13^ medical diagnosis^14^ and treatment selection.^15^ Standard applications of machine learning cannot work with information about data quality. However, in drug discovery, the presence of data heterogeneity has motivated techniques that explicitly account for varying quality in other ways.

Buterez et al.^16^ demonstrated significant model performance improvements by combining low-fidelity data from primary screens with high-fidelity data from confirmatory assays. Similar multifidelity approaches have been successfully applied to materials screening^17^and organic electronics property prediction.^18^ While these studies focus on varying data fidelities across different assay protocols, our approach examines the varying uncertainties within a single assay. This approach provides a continuous, rather than discrete, measure of data point quality and involves using a single model rather than combining separate models for different fidelities.

An alternative approach by Gutierrez et al.^19^ used a Gaussian process to model dose response outcomes directly using a multioutput model. This type of model can be used with any type of dose response data and they were able to identify the importance of different features on the two problems they tested. Because they do not use summary metrics to predict properties, they avoid the problem of unreliable metrics. This may be a drawback in some scenarios, as EC50s are standard metrics that are often used in drug discovery processes. The method also scales poorly as the number of samples and the number of doses tested increases. In contrast, our approach still follows the standard procedure of modeling EC50s but accounts for the varying quality of the data.

Rahman et al.^20^ addressed a different but related challenge: dose–response curves that do not fit to the standard Hill equation. Their solution introduced a functional random forest to model entire dose–response curves, enabling better experimental predictions. Our problem differs because we study curves that do conform to the Hill equation but lack sufficient experimental data for reliable fitting. In this context, modeling EC50s remains valuable as it provides a meaningful single metric for summarizing a molecule’s potential drug effectiveness.

The uncertainty of EC50 values has been studied by Watt and Judson,^21^ who used the bootstrap to estimate the uncertainty of values calculated via nonlinear regression. When detecting active molecules this system was able to identify false positives and false negatives and flag molecules with poor fits for manual inspection. Noise has been demonstrated to limit QSAR model performance^22^ and Kolmar and Grulke^7^ explored this effect by adding artificial noise to their data sets. They found that QSAR models can make predictions that contain less error than their training data and that the performance on error-laden test data can give a misleading estimate of model accuracy.

More generally, uncertainty in dose response data has been investigated in different domains. For example, in radiology, studies have examined how uncertainty in doses impacts models^23,24^ and toxicology research has explored the effect of extrapolating dose response data^25^ and using probabilistic approaches to fit models.^26^ Other research has looked at estimating QSAR model uncertainty^27^ and representing the uncertainty graphically so that it is easily understandable.^28^ These studies reflect the importance of understanding uncertainty within models, particularly when they are used to make decisions.

Cortes-Ciriano et al.^29^ analyzed how the presence of noise affected the performance of 12 different machine learning models across 12 bioactivity data sets. They found that model performance degraded across all models as noise increased, with gradient boosting machines showing particular sensitivity to noise. On certain data sets with high noise levels, support vector machines with a polynomial kernel and a Gaussian process with a polynomial kernel also demonstrated increased sensitivity to noise.

Collectively, these studies establish that bioactivity data sets contain inherent noise that can be quantified and significantly impacts machine learning model performance. Our work builds on these by introducing new methods for estimating uncertainties in input data and using these estimates in machine learning to mitigate the effect of epistemic uncertainty in EC50s on modeling.

In the next section we describe our methods to calculate EC50s (or IC50s for inhibition data sets) and quality metrics from dose response data. We then introduce our machine learning methods and experimental setup before presenting our results on both publicly available data from PubChem and data sets provided by BASF. The work finishes with a discussion of these results and their implications for improving predictive modeling in drug discovery.

## Data Preparation

The data sets we worked with consisted of a set of dose response experiments for each molecule. From these we calculated a summary metric; most of the data sets we worked with measured the efficacy of molecules, for these we calculated EC50s, the remaining data sets were inhibition data sets and for these we calculated IC50s. The process for these calculations is very similar, with the only difference being a shift in constant values for the inhibition data sets. For the rest of the methods section we describe the process for EC50s, but with IC50 values the process is the same.

We produced EC50s for machine learning in two ways, using a regression approach and a Bayesian analysis. The remainder of this section describes both processes. To get a set of molecular features from a SMILES string from PubChem, we use the RDkit Python package to produce a 1024-bit Morgan fingerprint.

Any molecules without recorded measurements were removed from the data set. We used all calculated EC50 values in our experiments, including those that had potentially unreliable fits. This allowed us to test whether our fit quality metrics could improve performance when a range of data qualities was present. We used all available experimental results (dose response pairs) during model testing.

### Regression Analysis

We converted dose response measurements to Hill equation parameters via nonlinear regression using the 2 parameter Hill equation:

$$\mathrm{Response}=\frac{100}{1+10^{\left({\mathrm{LogEC}}_{50}-X\right)\mathrm{HillSlope}}}$$ 1

We used this form because most of the experiments we worked with had responses that were between 0 and 100 so they could be adequately represented without using a more complex 4 parameter model. Some experimental results were outside this range, to handle these outliers we set results below 0 to 0 and results above 100 to 100. This meant that all values were predictable using the assumed form of the EC50 curve. We used the scipy curve fit package to select the model parameters that minimized the squared error of the model.^30^ In cases where there are not enough points to calculate the slope, we set the HillSlope parameter set to 1. This process follows the recommendations of Motulsky and Christopoulos.^10^

We then calculated the average squared error per dose–response experiment to get a metric for how well the curve fits the data, which we then used in the machine learning step as an estimate of the quality of the EC50 value. This process is illustrated graphically in Figure 2a. We show the raw dose–response measurements, the fitted curve, and the residuals whose average squared length gives the quality metric for the EC50 of that molecule.

**Figure 2:**
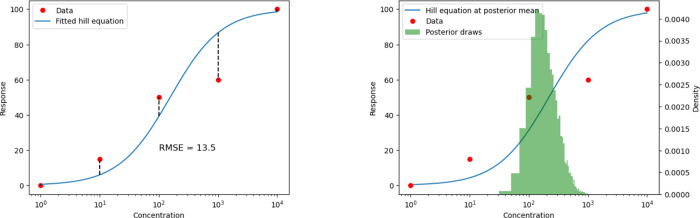
Graphical examples of regression and Bayesian fitting procedures.

### Bayesian Analysis

For the Bayesian analysis, we assumed that the noise in experimental measurements is normally distributed. This is a standard model for experimental noise in the absence of known systematic errors.^7^ To estimate the EC50 value and a quality metric, we drew samples from the posterior distribution and used the mean of these posterior samples as the target value for machine learning and their variance as a quality metric.

To estimate the noise present in the experiments, we used the pooled variance estimate with all dose–response experiments that had repeat measurements. For data sets where a pooled variance could not be calculated, we used the mean of the variance estimates for other data sets.

For the Bayesian analysis, we took uniform priors for the EC50 between 0.1 times the minimum experimentally tested concentration and 10 times the maximum experimentally tested concentration. This prior assumes the design of the experimental process selected reasonable concentration ranges. The prior for the slope parameter of the Hill equation was uniform between 0.1 and 10. This range encompasses all reasonable slope values.

To calculate the posterior distribution, we evaluated the posterior probability for pairs of EC50 and slope values using the two parameter Hill equation (eq 1) and handling results outside the range in the same way as during the regression analysis­(setting results below 0 to 0 and above 100 to 100). We did this for all pairs of 100 uniformly sampled EC50 values and 10 uniformly sampled slope values. From the resulting distribution, we drew 1000 samples to obtain a posterior sample. The mean of this sample was taken as the target value and the variance as a quality metric. This process is visualized in Figure 2b, using the same example dose–response data as Figure 2a. This figure displays the raw dose–response data alongside the resulting posterior density of EC50 values.

### Preparing Data for Machine Learning

In this study, our data contained unreliable EC50 values because, for some molecules, the experimental data did not fit the Hill equation well. As a result, evaluating models based on their ability to predict EC50 values may be misleading, as it would test their ability to replicate unreliable test set EC50s. To address this, we assessed models by predicting the outcomes of dose–response experiments directly. This allowed for a meaningful test on molecules for which a reliable EC50 could not be estimated and follows the suggestions of Kolmar and Grulke^7^: “QSAR models are being judged by their ability to predict error laden values, when they should be judged by their ability to predict the population means of measurements”.

As an example, if we had a molecule with only zero responses, then any EC50 prediction larger than the largest concentration tested would be reasonable. Using the standard approach, the curve-fitting procedure would choose one of many values that give zero error, and then we would judge our model on how well it selects this arbitrary value.

We compare our test procedure to the standard one graphically in Figure 3. The key difference is that in our approach we did not calculate EC50s for the test set and instead evaluated how well our model can predict actual experimental outcomes. The steps required in this process are

1. Randomly split data into a training and test set
2. Convert training set dose response values to EC50s using either regression analysis or Bayesian analysis
3. Use molecular descriptors, calculated EC50s and uncertainty values to build a model - see next section for details
4. Use the model to generate predicted EC50s for test set molecules
5. Predict outcomes of experiments in test set using calculated EC50s
6. Compare these predicted values to the experimental results

We used a Hill slope of one for each molecule during test time because we do not have information about the slope and one is the default slope value.^10^ This allowed for a consistent comparison of the utility of the EC50 value between models without having to model the slope values themselves.

**Figure 3:**
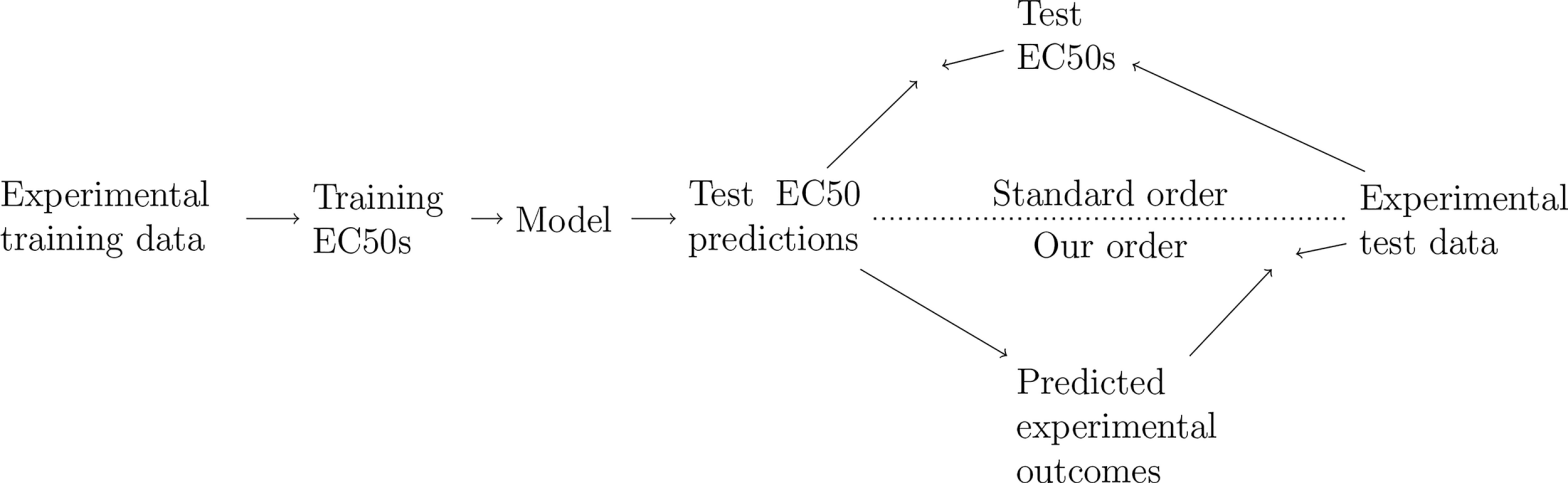
Schematic comparing the standard approach to our modified testing procedure. The difference is in which values are tested: in this study we test how well we can predict experimental values rather than estimated EC50 values. The point where arrows pointing in opposite directions meet is where the evaluation metric is calculated.

## Machine Learning Methods

This section gives an overview of the models used in this study. We used random forests, random forest variations and support vector regressors. These models were chosen over alternatives such as deep learning, boosting or Bayesian methods because they have been demonstrated to be the strongest models on bioactivity problems. A large scale comparison study on 2764 QSAR data sets found that random forests were the best model most often, performing best on 1535 data sets, and a support vector machine was best second most often, on 298 data sets.^11^ QSAR data sets are typically medium size (∼10 k samples) tabular data sets with high dimensionality­(∼1 k features). Under these conditions random forests are known to perform well and in particular outperform deep learning models.^31^

For further details of a standard random forest and a random forest with output smearing see Breiman^32^; for details of a forest with parametric bootstrap, a weighted forest and a variable output smearing random forest see Bellamy and King.^12^

### Random Forest Variations

We used the scikit learn^33^ package to build our random forests and always use 250 trees per forest. This number was chosen to be as large as possible while the experiments could still be run in a reasonable amount of time. The hyperparameter values not specifically mentioned in Table 1 were left at their default values.

**Table 1:** Parameter Values Used in Model Optimization^a^

parameter	values
*max_features*	0.1,0.33, 0.5, 1.0
*min_samples_split*	1%, 2.5%, 5%, 10%
α	0, 0.33, 1, 2
β	0.25, 1, 1.5, 2.5
*epsilon*	0.05, 0.1, 0.2, 0.5

a Possible *min_samples_split* values are given as a percentage of the total training data points so will change as dataset size changes. For example, if we had a training dataset of 500 points, the possible values will be 5, 13, 25, and 50.

#### Standard Random Forest

As a baseline model, we used a standard random forest regressor. Decision trees are ensembled with randomness added via the bootstrap and random feature selection.

#### Parametric Bootstrapping

The parametric bootstrap replaces the bootstrap step in a random forest. The bootstrap step in a random forest builds a data set for each tree by sampling *n* samples from the original *n* samples with replacement. In the bootstrap points are selected with equal probability. With the parametric bootstrap points are selected with a probability depending on their quality metric. To use this model, we transformed our quality metrics into a probability distribution using

$$p_{i}\propto e^{-\alpha z_{i}}$$ 2

Where *p*_*i*_ is the probability of that data point being selected in the bootstrap sample, *z*_*i*_ is the quality metric and α is a parameter that is used to control the impact of the quality metric on the model. Larger α values increase the frequency at which high quality data points are sampled in the bootstrap step. This will cause the model to favor fitting higher quality data points without ignoring the lower quality data points.

#### Weighted Forests

In a random forest, the splits of the individual decision trees are chosen by maximizing the variance reduction. In a weighted random forest the weighted variance reduction is maximized instead. As with a random forest with parametric bootstrap, weighting splits according to data quality causes the model to select splits in a way that favors fitting higher quality data points. In our experiments, the weights for each point were calculated using eq 2.

#### Output Smearing

An output smearing random forest replaces the random feature selection step of a random forest with smearing of output values. In each decision tree the target values have Gaussian noise added at random. This process serves as an alternative source of randomness for the forest. The variance of this Gaussian noise is a parameter that must be tuned. In our experiments it replaced the textitmax_features parameter of a standard forest.

#### Variable Output Smearing

In variable output smearing the amount each data point is smeared by depends on its quality metric - data points with higher quality are smeared less. This can improve performance by adding more randomness to the model for lower quality data points. Recent work has shown that randomness can regularize a random forest model^34^ and, by adding randomness selectively, this method may be able to adaptively regularize the model; fitting high quality data points well, while regularizing the model in noisy areas of the data set. In our implementation we added noise to each data point using

$$N\left(0,\;\frac{\beta }{p_{i}}\right)$$ 3

Where β is a parameter that controls the average amount of smearing across all data points and *p*_*i*_ values are from eq 2.

### Support Vector Regression

A support vector regressor was built using the scikit learn package. An *rbf* kernel is used and the *epsilon* parameter is optimized with other parameters left at their default values.

#### Weighted Support Vector Regression

In addition to a standard support vector regressor, we used a weighted support vector regressor which can incorporate quality information into the model.^35^ The weights for each point were calculated using eq 2. As with a weighted random forest, this method fits the model favoring high quality data points, giving them more impact on the final model.

### Experimental Procedure

We took real data sets for which we had dose response data and split them into training and test sets (PubChem data sets 75% train 25% test and BASF data sets 50% train and 50% test). A larger test set was used on the BASF data as these data sets were much larger, so fewer points were needed for a sufficient training set. On the training data, we calculated EC50 values and quality metrics as described in the methods sections. We then performed model optimization on all seven on the machine learning methods using random search cross validation. Random search was chosen as a search method as it is an efficient and easy to implement parameter search procedure.^36^ This random search procedure used the values shown in Table 1. These values represent standard parameter setups for these models.^11,12,32,37^ A model was then built, using the optimized parameters, on the full training set and used to predict the responses of test set dose response experiments. Models were evaluated on the root mean squared error of these predictions.

For each of our models we optimized parameters using random search with cross validation.^36^ At each search step parameters were selected at random from the possible values parameter values shown in Table 1. For the forests without output smearing we always optimize the *max_features* and *min_samples_split* parameters. For a parametric bootstrap and weighted forests we also optimize the α value (eq 2). With the output smearing forests we optimize the *min_samples_split* and the mean of the amount of output smearing added (β). For a variable output smearing forest we also optimize the smearing weight parameter (α in eq 2). For the support vector regressor we optimize the *epsilon* parameter and with the weighted version we also optimize the α value (eq 2) controlling the weights. We tested 20 randomly selected points for each data set and model type, using the model parameters with the smallest cross validation mean squared error to build the full model.

## Results

### PubChem Data Sets

We used 23 data sets from PubChem. These data sets were collected starting with the same 23 data sets as Buterez et al.^16^ From this set data sets were removed if suitable dose response measurements were not present (AIDs 1431, 1949, and 1259350), and some additional data sets were added (AIDs 1902, 602473, and 743254). Details of the data sets used, including PubChem AID and the original source of the data are given in Table 2.

**Table 2:** Sources for PubChem Datasets^a^

assay AID	original source	molecules
1902	Broad Institute	3227
2382	Broad Institute	2239
435010	Broad Institute	1797
449756	Broad Institute	1811
463203	Broad Institute	721
504941	Broad Institute	161
624273	Broad Institute	359
687027	Broad Institute	1024
720512	Broad Institute	109
1259418	Broad Institute	711
1259420	Broad Institute	174
493155	Burnham Center for Chemical Genomics	973
602473	Burnham Center for Chemical Genomics	2420
624326	Burnham Center for Chemical Genomics	985
624474	Burnham Center for Chemical Genomics	1327
1445	Southern Research Molecular Libraries Screening Center	655
1465	Southern Research Molecular Libraries Screening Center	980
449762	Southern Research Specialized Biocontainment Screening Center	1754
504329	Southern Research Specialized Biocontainment Screening Center	902
624330	Southern Research Specialized Biocontainment Screening Center	1570
504313	Emory University Molecular Libraries Screening Center	855
743254	The Scripps Research Institute Molecular Screening Center	254
1259375	The Scripps Research Institute Molecular Screening Center	348

a We give the PubChem AID for bioassays, the original source that provided the data to PubChem and the total number of molecules with dose response measurements in the assay.

The root mean squared error of predictions on test set dose response experiments are shown in Table 3 for nonlinear regression data analysis and Table 4 for Bayesian data analysis. We also calculate the standard deviation of these results using the bootstrap and show this in brackets.^38^ Results that are significantly better, using a Wilcoxon signed-rank test with a 0.1% significance level, than the equivalent method that does not use quality information are marked with an *. We used a low significance level to minimize the risk of type 1 errors­(rejecting the null hypothesis when it is true). For the random forest with parametric bootstrap and a weighted random forest the equivalent method is a standard random forest; for a variable output smearing random forest it is an output smearing random forest; and for a weighted support vector regressor it is a support vector regressor. Because the Wilcoxon test evaluates significance based on the ranks of paired performance differences rather than their absolute magnitudes, it is less sensitive to outliers than the root mean squared error (RMSE), making it possible for a model with a higher RMSE to be significantly better due to more consistent performance.^39^

**Table 3:** Root Mean Squared Error and Standard Deviation for Optimized Models on PubChem Datasets with Nonlinear Regression Data Analysis^a^

AID	RF	PB-RF	W-RF	OS	VOS	SVR	WSVR
1902	21.77(0.32)	21.71(0.33)	21.70(0.32)	20.75(0.29)	20.81(0.30)	20.24(0.30)	20.23(0.31)
2382	20.96(0.41)	20.96(0.40)	20.96(0.40)	20.77(0.39)	20.77(0.38)	20.84(0.38)	20.48*(0.42)
435010	21.41(0.37)	21.38(0.38)	21.42(0.38)	21.34(0.37)	21.34(0.38)	21.77(0.35)	21.69*(0.38)
449756	27.26(0.30)	26.88*(0.30)	26.89*(0.30)	26.99(0.30)	26.93(0.30)	27.34(0.30)	27.34(0.31)
463203	12.54(0.30)	12.38*(0.32)	12.43*(0.31)	12.84(0.30)	12.85(0.30)	12.83(0.27)	12.06*(0.31)
504941	12.40(0.57)	12.08(0.58)	12.10(0.55)	12.27(0.55)	12.25(0.55)	13.08(0.54)	13.08(0.53)
624273	15.28(0.54)	15.24(0.55)	15.25(0.53)	15.26(0.50)	15.28(0.53)	15.39(0.54)	15.26*(0.50)
687027	29.74(0.59)	29.74(0.58)	29.74(0.59)	29.36(0.58)	29.36(0.59)	28.83(0.58)	28.78(0.58)
720512	7.99(0.24)	8.00*(0.24)	7.98*(0.23)	7.96(0.24)	7.95(0.24)	7.83(0.23)	7.85(0.23)
1259418	12.56(0.28)	12.44(0.29)	12.42(0.28)	12.42(0.28)	12.43(0.28)	12.76(0.27)	12.76(0.28)
1259420	13.26(0.61)	13.33(0.63)	13.32(0.63)	13.43(0.61)	13.44(0.61)	13.63(0.61)	13.47(0.58)
493155	11.68(0.19)	11.63(0.19)	11.59(0.19)	11.63(0.19)	11.55*(0.19)	11.89(0.19)	11.82*(0.20)
602473	21.71(0.33)	20.53*(0.34)	20.30*(0.33)	21.24(0.34)	21.35(0.33)	21.16(0.30)	21.28*(0.30)
624326	14.24(0.20)	14.15*(0.20)	14.23(0.20)	14.3(0.21)	14.28(0.20)	14.54(0.19)	14.52(0.19)
624474	19.77(0.31)	19.77(0.32)	19.77(0.33)	19.10(0.32)	19.10(0.32)	18.77(0.32)	18.56*(0.31)
1445	16.77(0.30)	16.51(0.31)	16.51(0.30)	16.88(0.31)	16.88(0.31)	17.69(0.32)	16.75*(0.30)
1465	7.31(0.39)	7.31(0.38)	7.30(0.37)	7.26(0.36)	7.27(0.36)	8.52(0.27)	7.34*(0.35)
449762	29.91(0.30)	29.53(0.31)	29.56(0.31)	29.63(0.31)	29.99(0.29)	29.55(0.31)	29.28(0.30)
504329	19.09(0.31)	18.52*(0.31)	18.52*(0.33)	16.73(0.32)	16.73(0.32)	18.08(0.32)	18.13(0.30)
624330	10.17(0.19)	10.18(0.20)	10.17(0.20)	9.93(0.19)	9.94(0.19)	9.95(0.18)	9.64*(0.18)
504313	7.35(0.18)	7.33(0.17)	7.37(0.18)	7.56(0.2)	7.54(0.19)	8.56(0.21)	8.56(0.21)
743254	18.27(0.45)	18.33*(0.44)	18.16*(0.43)	18.06(0.47)	18.21(0.45)	18.32(0.45)	18.35(0.44)
1259375	8.33(0.36)	8.28(0.37)	8.33(0.37)	8.29(0.36)	8.32*(0.36)	8.25(0.35)	8.19*(0.34)

a Results with an * show that the results are significantly better than the equivalent model without quality information. RF, random forest; PB-RF, random forest with parametric bootstrap; W-RF, weighted random forest; OS, random forest with output smearing; VOS, random forest with variable output smearing; SVR, support vector regression; WSVR, weighted support vector regression.

**Table 4:** Root Mean Squared Error and Standard Deviation for Optimized Models on PubChem Datasets with Bayesian Data Analysis^a^

AID	RF	PB-RF	W-RF	OS	VOS	SVR	WSVR
1902	21.81(0.31)	21.78(0.33)	21.76(0.32)	20.71(0.31)	20.76(0.30)	20.53(0.29)	20.53(0.31)
2382	21.28(0.38)	21.28(0.38)	21.28(0.38)	21.11(0.39)	21.11(0.37)	22.38(0.33)	20.48*(0.40)
435010	21.96(0.37)	21.95(0.37)	21.92*(0.39)	21.85(0.36)	21.86(0.38)	22.94(0.37)	22.94(0.38)
449756	27.66(0.30)	26.91*(0.29)	26.95*(0.29)	27.04(0.29)	27.11(0.29)	27.38(0.29)	27.39(0.31)
463203	12.97(0.30)	12.77*(0.31)	12.75*(0.31)	13.20(0.31)	13.19(0.31)	14.35(0.26)	12.35*(0.31)
504941	12.49(0.53)	12.52(0.55)	12.51(0.55)	12.59(0.53)	12.60(0.54)	13.64(0.49)	13.64(0.49)
624273	15.37(0.53)	15.37(0.51)	15.35(0.56)	15.39(0.53)	15.35(0.52)	16.36(0.50)	16.36(0.48)
687027	29.55(0.60)	29.09*(0.60)	29.09*(0.58)	28.79(0.59)	28.79(0.58)	29.10(0.60)	28.69*(0.59)
720512	7.92(0.19)	7.93(0.20)	7.93(0.19)	7.90(0.20)	7.92(0.20)	8.60(0.19)	8.07(0.18)
1259418	12.80(0.31)	12.80(0.30)	12.80(0.28)	12.70(0.30)	12.70(0.30)	12.84(0.29)	12.84(0.29)
1259420	13.22(0.60)	13.20(0.58)	13.20(0.61)	13.29(0.59)	13.29(0.61)	13.76(0.64)	13.51(0.63)
493155	11.53(0.18)	11.63(0.19)	11.57*(0.18)	11.62(0.19)	11.60(0.19)	11.86(0.19)	11.78*(0.20)
602473	21.69(0.35)	20.00*(0.31)	20.20*(0.30)	21.19(0.32)	21.17*(0.33)	22.28(0.28)	22.26*(0.27)
624326	14.33(0.19)	14.31(0.20)	14.30(0.20)	14.59(0.21)	14.50*(0.20)	14.49(0.20)	14.48(0.19)
624474	20.19(0.31)	20.23(0.30)	20.23(0.31)	19.46(0.29)	19.46(0.31)	19.17(0.30)	18.89*(0.30)
1445	18.74(0.33)	18.74(0.33)	18.74(0.33)	18.82(0.34)	18.82(0.32)	18.86(0.31)	18.86(0.32)
1465	7.68(0.33)	7.68*(0.34)	7.68*(0.34)	7.65(0.33)	7.66(0.34)	12.43(0.23)	7.59*(0.32)
449762	30.67(0.30)	30.61(0.31)	30.66(0.30)	30.48(0.30)	30.49(0.29)	30.72(0.30)	30.56(0.31)
504329	18.93(0.31)	18.93(0.31)	18.93(0.32)	16.95(0.32)	16.95(0.31)	18.95(0.31)	18.95(0.29)
624330	11.58(0.20)	11.47*(0.20)	11.47*(0.20)	11.24(0.21)	11.22(0.18)	10.75(0.18)	10.64*(0.18)
504313	6.95(0.15)	6.94*(0.15)	6.94*(0.16)	6.93(0.15)	6.93(0.15)	7.71(0.17)	7.71(0.17)
743254	18.34(0.48)	17.95*(0.46)	18.18(0.47)	17.81(0.45)	17.78(0.46)	18.21(0.46)	18.21(0.46)
1259375	8.39(0.38)	8.39(0.35)	8.33(0.37)	8.41(0.37)	8.29(0.37)	8.34(0.35)	8.26*(0.36)

a Results with an * show that the results are significantly better than the equivalent model without quality information. RF, random forest; PB-RF, random forest with parametric bootstrap; W-RF, weighted random forest; OS, random forest with output smearing; VOS, random forest with variable output smearing; SVR, support vector regression; WSVR, weighted support vector regression.

On nonlinear regression data, Table 3, the performance difference between optimized models was generally small, but there were some larger improvements and many significant improvements in performance. A modest performance improvement was expected on these data sets, as the variation in quality between data points was generally small: data points usually had the same number of measurements at the same concentrations, leading to only small variations in quality between curve-fits.

The degree of success with which different machine learning methods were able to use uncertainty information appears to be linked to the data set’s original source. For example, the weighted random forest and weighted support vector regressor were both significantly better than a standard random forest on 3 of the 11 data sets from the Broad institute. But, on the 9 data sets from the Burnham center for chemical genomics and the Southern research screening centers weighted random forests were significantly better on 2 data sets but weighted support vector regressors had a significant improvement on 6 data sets. This seems to indicate that there are experimental design features which influence how well different methods can utilize the corresponding quality information. Investigating this observation further could be an interesting area for future work.

With Bayesian data analysis, Table 4, performance was similar to that with nonlinear regression analysis, with generally small differences between models. With Bayesian analysis the weighted support vector regressor and variable output smearing forest performed significantly better less often. However, the weighted random forest and weighted support vector regressor performed better more often. This indicates a link between the form of the uncertainty information and how it was used by the model. In Figure 4 we summarize how often using each kind of data analysis led to better, the same or worse performance for the four methods that incorporated quality information.

**Figure 4:**
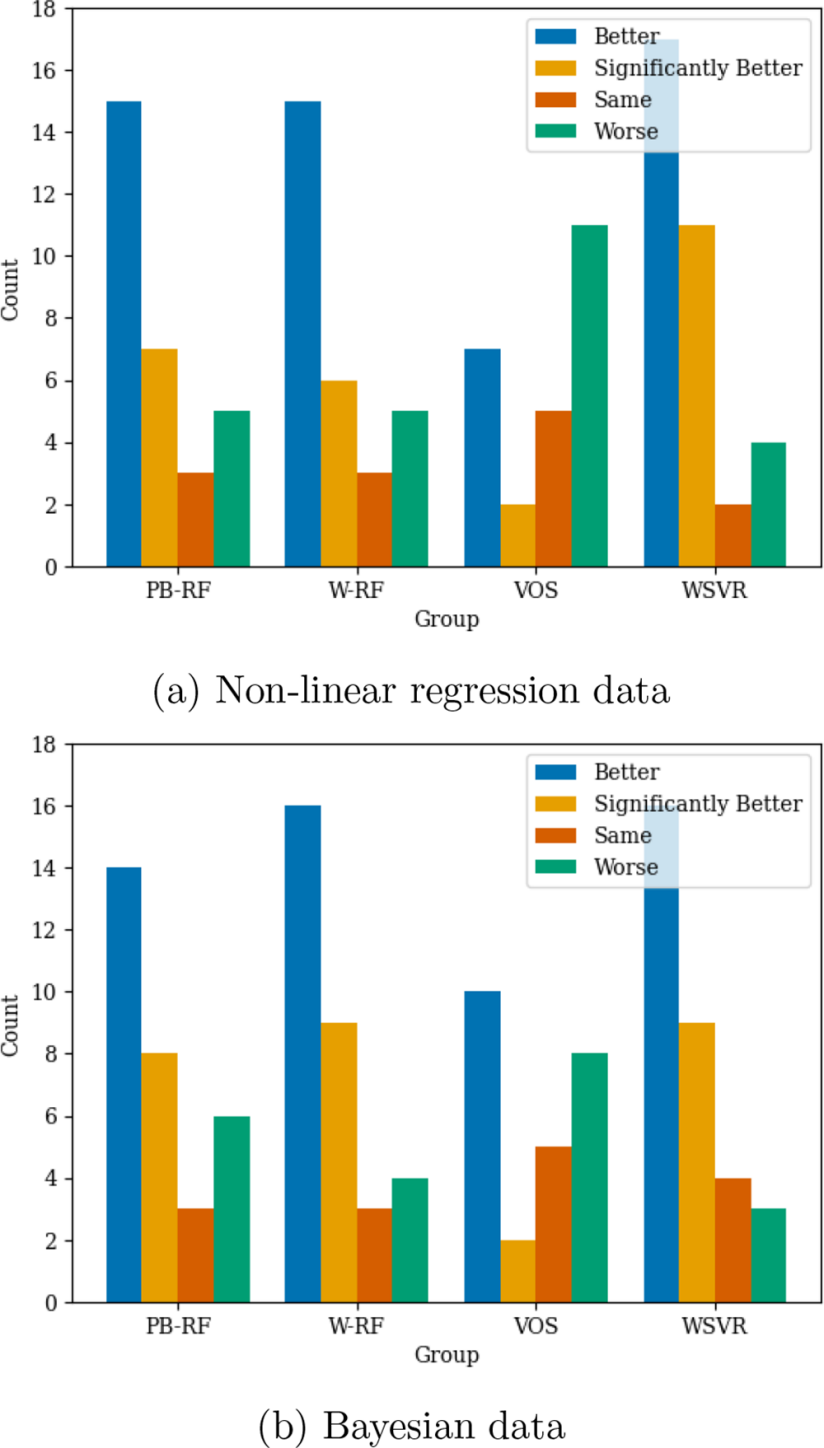
Number of times uncertainty information caused model performance to be better, significantly better, the same and worse, than the equivalent model that did not use this information on the PubChem data sets. PB-RF, random forest with parametric bootstrap; W-RF, weighted random forest; VOS, random forest with variable output smearing; SVR, WSVR, weighted support vector regression.

The summary in Figure 4 shows that with nonlinear regression data analysis the random forest with parametric bootstrap, weighted random forest, and weighted support vector regressor were all strong methods. Variable output smearing performed worse, being better on less than half the data sets and significantly better only twice. A similar pattern was seen with Bayesian data analysis. In Table 5 we show how often a model was the best performing model on a data set. The first two rows show the best performers for each type of data analysis. In the third row, we show the best performer overall, with the two values in each row showing the number of times the relevant model performed best with nonlinear regression analysis (first value) and Bayesian analysis (second value). Because we predicted dose response values, the test set does not change between data analysis methods, so we can make a comparison between them.

**Table 5:** Number of Times a Model Had the Best Performance on the PubChem Datasets^a^

data analysis	RF	PB-RF	W-RF	OS	VOS	SVR	WSVR
nonlinear regression	1	1	2	4	2	1	8
Bayesian	3	3	2	5	2	0	8
overall	0,1	5,2	0,0	3,1	1,1	1,0	7,1

a For the overall row, the first number is the number of times the method with nonlinear regression analysis was best and the second is when the method with Bayesian analysis was best. For example, the 5,2in the PB-RF column means that on 5 PubChem datasets the best test set performance was from a PB-RF model trained on nonlinear regression data and on 2 datasets it was a PB-RF model trained on the data with Bayesian analysis.

The weighted support vector regressor was the best method most often in all rows of Table 5. However, overall forest based methods are best more often the than support vector methods. This result is in agreement with other results in the field^11^ and it is reassuring to see that the relative performance of machine learning methods was similar when predicting dose response experiments directly instead of EC50s. In general, the best performing model was one trained on data analyzed using nonlinear regression but, on 6 of the 23 data sets, we get better model performance using the Bayesian analysis.

### BASF Data Sets

As part of an ongoing research collaboration, BASF provided a collection of 17 data sets from pesticide design assays. These data sets differ from the PubChem ones because they are larger, have noisier measurements and the number of measurements for each molecule varies much more. For example, some molecules have over 100 experiments whereas some have fewer than 10. This large difference in number of measurements means that the range of data point quality is much greater within the BASF data sets. In Table 6 we show the number of molecules in each data set.

**Table 6:** Number of Molecules Present in Each of the BASF Datasets

BASF ID	molecules
1	28,017
2	27,294
3	26,875
4	23,263
5	15,386
6	11,080
7	10,911
8	6662
9	6653
10	2522
11	2246
12	1982
13	1955
14	1545
15	1487
16	1343
17	1180

In Table 7 we show the results on nonlinear regression data and in Table 8 we show the results for Bayesian data. As in the previous section we denote when using quality information led to a significant performance improvement, using a Wilcoxon signed-rank test at a 0.1% significance level.

**Table 7:** Root Mean Squared Error and Standard Deviation for Optimized Models on BASF Datasets with Nonlinear Regression Data Analysis^a^

ID	RF	PB-RF	W-RF	OS	VOS	SVR	WSVR
1	28.17(0.07)	27.79*(0.07)	27.76*(0.07)	27.80(0.08)	27.79(0.07)	24.94(0.07)	24.90*(0.08)
2	26.84(0.08)	25.33*(0.08)	25.36*(0.07)	27.21(0.08)	27.18*(0.08)	23.81(0.08)	23.50*(0.08)
3	36.78(0.08)	28.64*(0.07)	28.64*(0.08)	36.43(0.08)	36.43(0.08)	29.21(0.06)	27.44*(0.07)
4	30.26(0.07)	30.26(0.07)	30.26(0.07)	29.83(0.07)	29.83(0.07)	29.29(0.06)	29.27*(0.06)
5	34.20(0.11)	33.66(0.10)	33.72(0.10)	32.86(0.10)	32.86(0.11)	30.20(0.1)	30.27*(0.11)
6	26.78(0.11)	26.74*(0.12)	26.61*(0.12)	26.80(0.11)	26.72(0.12)	26.52(0.09)	23.97*(0.13)
7	26.71(0.11)	26.71(0.12)	26.71(0.12)	26.45(0.12)	26.45(0.11)	24.66(0.12)	24.18*(0.13)
8	28.73(0.14)	28.73(0.14)	28.73(0.14)	28.38(0.15)	28.38(0.15)	28.36(0.12)	27.30*(0.14)
9	35.23(0.13)	35.23(0.13)	35.23(0.13)	34.64(0.13)	34.64(0.13)	31.78(0.11)	30.00*(0.13)
10	36.57(0.26)	35.26*(0.25)	35.37*(0.25)	36.07(0.26)	36.07(0.24)	34.97(0.26)	34.26*(0.25)
11	36.09(0.28)	35.41*(0.27)	35.39*(0.27)	35.00(0.28)	35.15(0.28)	35.03(0.29)	35.14(0.28)
12	33.97(0.28)	33.99(0.29)	34.00(0.28)	33.22(0.28)	33.19(0.29)	33.38(0.30)	33.62(0.28)
13	33.75(0.27)	33.84(0.26)	33.76(0.28)	32.94(0.27)	32.95(0.27)	32.26(0.28)	32.27(0.26)
14	34.42(0.22)	34.73(0.24)	34.71(0.22)	39.58(0.25)	39.06*(0.24)	44.82(0.26)	44.82(0.27)
15	32.32(0.35)	32.24*(0.36)	32.33(0.37)	31.54(0.37)	31.60(0.37)	31.09(0.34)	31.19(0.34)
16	15.79(0.42)	15.47*(0.43)	15.47*(0.41)	15.58(0.43)	15.57(0.40)	15.39(0.43)	15.59(0.40)
17	32.47(0.40)	31.97*(0.41)	31.91*(0.38)	32.02(0.40)	32.00(0.38)	30.94(0.36)	31.01(0.37)

a Results with an * show that the results are significantly better than the equivalent model without quality information. RF, random forest; PB-RF, random forest with parametric bootstrap; W-RF, weighted random forest; OS, random forest with output smearing; VOS, random forest with variable output smearing; SVR, support vector regression; WSVR, weighted support vector regression.

**Table 8:** Root Mean Squared Error and Standard Deviation for Optimized Models on BASF Datasets with Bayesian Data Analysis^a^

ID	RF	PB-RF	W-RF	OS	VOS	SVR	WSVR
1	28.86(0.06)	29.22(0.06)	28.62*(0.07)	28.02(0.07)	28.05(0.07)	26.05(0.07)	26.01*(0.07)
2	27.75(0.07)	28.00(0.06)	26.51*(0.07)	28.12(0.07)	28.10(0.07)	25.98(0.06)	25.25*(0.07)
3	36.61(0.07)	36.56(0.07)	29.41*(0.07)	36.95(0.07)	36.95(0.07)	32.65(0.05)	29.64*(0.06)
4	30.65(0.07)	30.87(0.07)	30.65(0.07)	30.15(0.07)	30.15(0.07)	33.37(0.05)	33.80(0.05)
5	33.05(0.09)	34.26(0.09)	32.94*(0.09)	32.25(0.10)	32.29(0.10)	30.39(0.09)	30.38(0.10)
6	26.60(0.11)	26.78(0.10)	26.64*(0.10)	26.78(0.10)	26.78(0.10)	28.67(0.08)	24.55*(0.11)
7	27.09(0.12)	27.31(0.11)	27.09(0.11)	27.00(0.12)	27.00(0.12)	26.85(0.11)	25.74*(0.12)
8	29.16(0.14)	29.45(0.14)	29.16(0.14)	28.94(0.14)	28.94(0.14)	31.33(0.10)	28.28*(0.13)
9	36.58(0.12)	36.66(0.13)	36.58(0.12)	36.12(0.12)	36.12(0.12)	35.53(0.10)	31.79*(0.11)
10	35.75(0.25)	35.79(0.26)	35.77(0.26)	35.92(0.25)	35.97(0.25)	36.34(0.24)	35.78*(0.26)
11	36.02(0.29)	36.20(0.27)	36.13(0.28)	35.91(0.28)	36.02(0.28)	37.18(0.30)	37.18(0.30)
12	34.69(0.28)	34.62(0.28)	34.63(0.29)	33.66(0.29)	33.65(0.29)	33.88(0.28)	33.96(0.29)
13	33.49(0.26)	33.32*(0.26)	33.50(0.27)	32.54(0.27)	32.48(0.27)	32.68(0.26)	32.77(0.26)
14	32.94(0.23)	34.65(0.23)	33.17(0.23)	31.41(0.24)	31.43(0.24)	44.10(0.25)	44.10(0.26)
15	32.85(0.35)	32.85(0.35)	32.92(0.36)	31.95(0.35)	31.97(0.36)	32.34(0.31)	32.53(0.31)
16	16.16(0.41)	16.31(0.43)	16.20(0.40)	16.56(0.42)	16.59(0.42)	16.78(0.37)	16.78(0.37)
17	33.13(0.38)	32.63*(0.36)	32.61*(0.40)	31.96(0.37)	31.89*(0.35)	32.62(0.32)	32.79(0.33)

a Results with an * show that the results are significantly better than the equivalent model without quality information. RF, random forest; PB-RF, random forest with parametric bootstrap; W-RF, weighted random forest; OS, random forest with output smearing; VOS, random forest with variable output smearing; SVR, support vector regression; WSVR, weighted support vector regression.

The results for BASF data with nonlinear regression data analysis had similar patterns to the results on PubChem data but, when uncertainty information was useful, it tended to lead to bigger performance improvements. The greatest improvement was on data set 3, where the RMSE dropped from 36.78 with a standard random forest to 28.64 with a weighted random forest. These larger performance differences were probably due to greater variation in data point quality, resulting in more benefit gained by accounting for it.

The results with Bayesian analysis, shown in Table 8, were weaker than the results on nonlinear regression data. The weighted support vector machine still performed well, but the other methods that use quality information all cause a decrease in performance at least as often as they cause an increase. On these methods there were again some large improvements in performance - for example, the weighted random forest on data set 3, the RMSE goes from 36.61 to 29.41 and on data set 2 the RMSE goes from 27.75 to 26.51. This shows that, while the Bayesian quality information was useful less often, it could still be utilized to give large performance improvements on some data sets. We summarize the number of times that using quality information led to better, significantly better, the same, and worse performance in Figure 5. Better and worse performance are defined using the root mean squared error of the test set.

**Figure 5:**
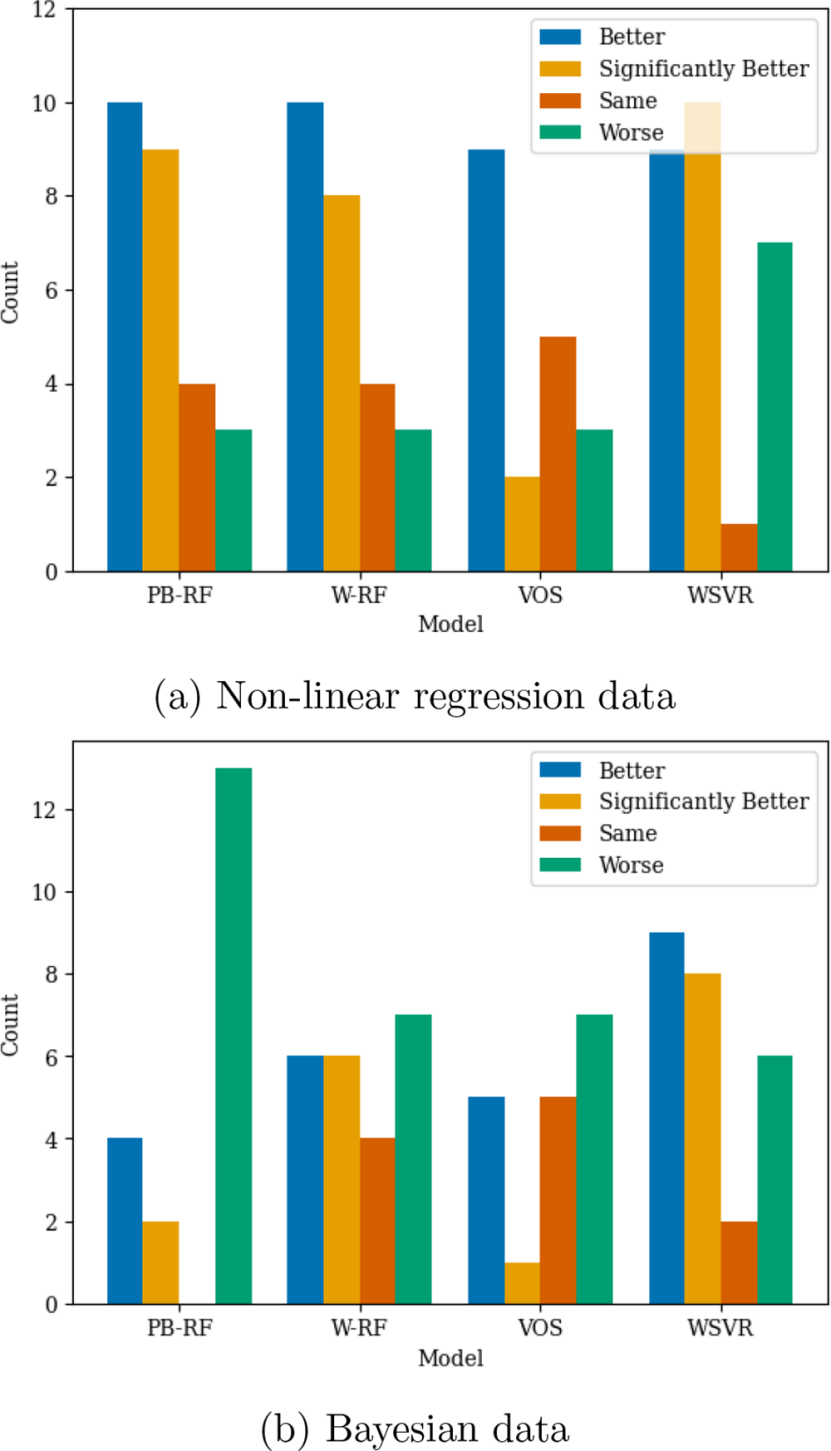
Number of times uncertainty information caused model performance to be better, significantly better, the same and worse, than the equivalent model that did not use this information on the BASF data sets. PB-RF, random forest with parametric bootstrap; W-RF, weighted random forest; VOS, random forest with variable output smearing; SVR, WSVR, weighted support vector regression.

From the summarized results in Figure 5, we see that the random forest with parametric bootstrap and weighted random forests were strong method with nonlinear regression analysis; they performed better most often and worse least often. However, with Bayesian analysis it performed worse most often. The opposite is true for the weighted support vector regressor, it performed worse most often with nonlinear regression analysis and least often with Bayesian analysis. We show the number of times each method was the strongest performer on nonlinear regression data, Bayesian data and overall in Table 9.

**Table 9:** Times a Model Has the Best Performance on the BASF Datasets^a^

data analysis	RF	PB-RF	W-RF	OS	VOS	SVR	WSVR
nonlinear regression	1	0	0	1	1	5	9
Bayesian	2	0	1	4	3	0	7
overall	0,0	0,0	0,0	1,1	1,0	5,0	9,0

a For the overall row, the first number is the times the method with nonlinear regression analysis was best and the second is when the method with Bayesian analysis is best.

The best performing method on the BASF data sets was almost always a support vector based method on nonlinear regression data. With Bayesian data, output smearing methods are also strong. The best method overall was, on all but two of the 17 data sets, trained with nonlinear regression data. These results differ from the PubChem ones, where support vector methods were only best about half the time and Bayesian data led to better performance on 6 of the 23 data sets, a much larger fraction than the 2 out of 17 on the BASF data. This demonstrates that the best method for data analysis, modeling and using quality information is data set dependent although nonlinear regression data analysis, weighted random forests and weighted support vector machines are consistently strong.

## Discussion

### Learning on Dose Response Data

By predicting dose response experiment outcomes we have been able to compare the effectiveness of a nonlinear regression data analysis to a Bayesian one. Our results show that the choice of method usually only makes a small difference and the best performance is normally obtained using the standard procedure of nonlinear regression. This difference between the data analysis methods also compares the effectiveness of the two different methods for generating quality metrics; mean curve fit error, and posterior variance. The simple quality metric of mean curve error performs well, causing a greater average performance improvement when used than the posterior variance from the Bayesian analysis: for the PubChem data sets using standard random forests, on average the RMSE was 1.7% lower with regression rather than Bayesian analysis and on weighted random forests the RMSE was 1.9% lower with regression analysis. This pattern was reversed in the support vector methods, where the regression method had an RMSE 5% lower for SVR and 2.5% lower for WSVR. In all cases the performance is better with regression analysis, but for random forest methods the use of quality information increases this difference, while for support vector methods it decreases it. This shows that for forest methods the regression quality information is more useful than the Bayesian quality information, while, for support vector methods this is reversed. A final advantage of the regression analysis is that it is less complex than the Bayesian analysis.

The random forest model is known to be a strong baseline for QSAR data sets.^11^ In Tables 5 and 9 we see that while forest methods often performed well, support vector methods were competitive and on the BASF data sets actually performed better. On the PubChem data, the strong performance of support vector methods was largely because the weighted support vector machine­(WSVR) was able to use quality information more effectively that its forest based counterparts. The mean RMSE of support vector regressor­(SVR) models was 0.9% greater than a standard random forest but a WSVR model had an RMSE 0.6% lower (1.6% lower than SVR). In comparison, a weighted random forest had an RMSE on average 0.8% lower than a standard random forest. The percentage performance improvement from incorporating quality information in support vector methods was double that of forest methods. This suggests that support vector methods are worth considering for QSAR problems when quality information is available, particularly for certain types of data sets, as shown by their strong performance on BASF data. These results also highlight that random forests benefit less from quality information, likely because they are naturally robust to noise.^34^ As a result, accounting for heteroscedastic noise in the data offers less advantage for forests compared to other models. This noise robustness may partly explain random forests’ strong performance on dose–response data and incorporating data quality information into other model types could make these methods more competitive on dose–response tasks.

### Utility of Uncertainty Information

Our results demonstrate that uncertainty information can improve model performance on dose response data across different data sets and model types, but on some data sets the quality information is not helpful. The likely reason for this is that data quality is relatively uniform (all data points have similar levels of uncertainty) between data points. In this case incorporating uncertainty information will not significantly improve performance, as the best approach would be to treat all data points equally, as in standard approaches. It can be achieved in our methods by setting the parameter α (eq 2) to 0. This means that if quality information is not useful, hyperparameter optimization can still get optimal performance. However, with random search optimization, attempting to use quality information when it is not relevant can degrade performance. This is because if a fixed number of iterations are used, adding an additional search dimension - such as incorporating uncertainty information - makes it harder to find good parameters if that dimension does not impact model performance. The size of the search space increases without the possibility of finding better parameters. This contrasts with the results on the random forest variations in Bellamy and King,^12^ which used grid search for optimization. Grid search requires more time for optimizing methods with uncertainty information, as a larger number of models need to be evaluated. However, performance never degrades as the best model is always identified — meaning that if quality information is not useful, it is not used.

### Parameter Selection

The methods we use to incorporate uncertainty information require an extra hyperparameter to be selected. An appropriate value is important to ensure strong performance. Figure 6 shows the change in the root mean squared error when this parameter­(α) is changed for the four methods that used uncertainty information on the PubChem data set AID 449756. For a random forest with the parametric bootstrap, the value of α was important, the performance varying as it increased. The weighted forest had an initial performance improvement followed by a relatively steady performance. For output smearing, the parameter also caused larger changes to performance. Finally, the performance of the weighted support vector regressor improved very slowly as the parameter increased. These patterns are the same as reported by Bellamy and King^12^ and we refer the reader to this work for further details on the models, including relative performance differences.

**Figure 6:**
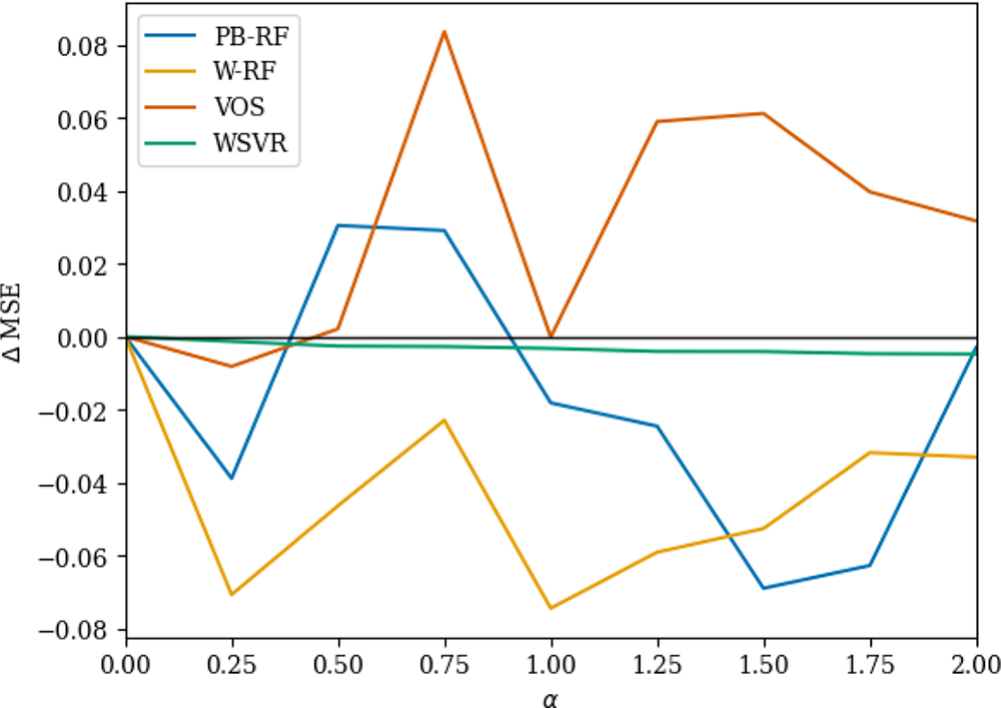
Change in root mean squared error as α is changed on data set AID 449756.

### Output Smearing Methods

Our results show that output smearing and variable output smearing generally perform worse than other methods. Although they occasionally yield the best model performance, this occurs less often than with other approaches, and variable output smearing consistently struggles to leverage uncertainty information effectively. Bellamy and King^12^ demonstrated that variable output smearing can be effective when data point quality depends on output value. This was expected for the BASF data sets, as their assays tend to focus on promising candidates, which increases the data quality of highly active molecules. Our results somewhat support this, as variable output smearing on the BASF data sets with nonlinear regression data analysis performed closer to other methods, improving performance on nine data sets—only one fewer than a random forest with parametric bootstrap and on par with other methods. However, it still performed significantly better less often than other methods. This relatively weak performance, even on data sets expected to benefit from variable output smearing, suggests that variable output smearing random forests are not very effective for modeling dose–response data.

### Limitations and Future Work

Our approach requires that raw experimental data is available, not just the summary metrics produced by curve fitting. This is a limitation, as often data sets are shared without full details of the experiments. Additionally, for this approach to be useful, there must be a range of data qualities present. A well designed study, that tests all molecules the same number of times over a reasonable concentration range, will give a data set with a uniform level of quality. In these cases, our approach will not have an advantage over standard approaches and may lead to worse performance.

Across our experiments, data sets from the same sources had a similar relative performance between methods. The reasons for this could be explored in future work, particularly looking at adapting methods based on assay features to get more consistent performance improvements. Other areas for future work could be to extend the proposed approach to different model types or different data set types. Investigating other types of model could be interesting because our results demonstrate that the benefit of quality information is not constant between models. Testing different types of machine learning models with quality information, including those typically less suited to drug design data, could result in strong performance. Our approach could also be extended to work with other types of data. If the quality of points varies throughout a data set and if these qualities can be estimated, then our approach can be used. This will be applicable to any problem where raw data are summarized via a curve fitting procedure before machine learning - as is the case in EC50 calculations.

## Conclusions

We have demonstrated that uncertainty information implicitly available in dose–response data can be extracted and used within QSAR models to improve predictions. This study, which compared two data analysis methods and seven machine learning models across 40 data sets, found that the use of quality information significantly improved performance on 31 of these data sets. For the data sets we analyzed, nonlinear regression with average squared error as a quality metric outperformed calculating a Bayesian posterior for Hill equation parameters with posterior variance as the quality metric. All machine learning methods tested showed improved performance, with the weighted random forest and weighted support vector regressor demonstrating particularly strong overall performance. These performance improvements are attainable without additional experimental data collection or altering upstream processes, adding only minimal computational overhead. Our approach is broadly applicable and can aid in developing more accurate models. These improved models can help researchers make informed decisions and enhance practical applications of QSAR models such as active learning strategies.

## Data Availability

Code for the results is available on GitHub­(https://github.com/hugobellamy/JCIM-DoseResponseQuality). PubChem data can be accessed on PubChem at ‘https://pubchem.ncbi.nlm.nih.gov/bioassay/’ + AID, where AID is the assay AID given in Table 2. The BASF data sets are not publicly available.
